# Objective Physical Activity and Sedentary Behaviour Patterns Among Informal Carers in the BCS70 Cohort

**DOI:** 10.3390/ijerph23020242

**Published:** 2026-02-14

**Authors:** Eilidh Russell, Alison Kirk, Mark D. Dunlop, Dwight C. K. Tse, Kieren Egan

**Affiliations:** 1 Department of Computer and Information Sciences, University of Strathclyde, Glasgow G1 1XH, UK; mark.dunlop@strath.ac.uk (M.D.D.); kieren.egan@strath.ac.uk (K.E.); 2 School of Psychological Sciences and Health, University of Strathclyde, Glasgow G1 1XQ, UK; alison.kirk@strath.ac.uk (A.K.); dwight.tse@strath.ac.uk (D.C.K.T.)

**Keywords:** physical activity, sedentary behaviour, caregivers, Great Britain, secondary data analysis, cohort study

## Abstract

**Highlights:**

**Public health relevance—How does this work relate to a public health issue?**

- This work addresses a public health issue by highlighting extremely low adherence to the UK Chief Medical Officers’ Physical Activity Guidelines among informal carers, a large and essential population whose lack of physical activity or prolonged sedentary behaviour increases their risk of chronic disease, poor mental health and overall wellbeing.
- By providing objective, population-level evidence of carers’ physical activity and sedentary behaviour patterns, this study can inform prevention strategies and tailored interventions to protect carers’ health and sustain health and social care systems

**Public health significance—Why is this work of significance to public health?**

- Informal carers are a large, essential population supporting and underpinning health and social care systems, this work highlights that they have extremely low adherence to physical activity guidelines, placing them at an increased risk of poor mental and physical health outcomes.
- This study identifies carers as a priority group for targeted public health interventions to reduce inactivity, protecting carer wellbeing whilst supporting and sustaining wider healthcare systems.

**Public health implications—What are the key implications or messages for practitioners, policy makers and/or researchers in public health?**

- Informal carers should be recognised as a priority group in public health policy and practice, with flexible, tailored physical activity interventions that account for time constraints, caregiving demands, and wellbeing needs.
- Researchers and policymakers should use objective monitoring and richer contextual data on caring roles to design, target, and evaluate interventions that can improve carers’ physical activity, health, and long-term sustainability of care.

**Abstract:**

While the health benefits of physical activity (PA) and reduced sedentary behaviour (SB) are well established, informal carers remain an under-researched group. Despite being known to face many barriers to PA, informal carers’ activity levels remain unclear due to mixed findings from previous research. Specifically, objective PA and SB levels of informal carers in Great Britain are currently unknown. The aim of this study was to examine PA and SB among informal carers using accelerometer data from the ‘Age 46’ Survey of the 1970 British Cohort Study (BCS70). Analyses of Covariance and Logistic Regressions were performed to: (i) compare carers’ and non-carers’ PA and SB, (ii) examine the impact of caring hours on PA and SB, and (iii) identify predictors of adherence to the UK Chief Medical Officers’ PA guidelines. After adjusting for covariates, (i) no differences were observed in PA or SB outcomes between carers and non-carers (*p* > 0.05) (e.g., mean daily step count 9316.06 vs. 9554.11 and mean sitting time 1.09 h/day vs. 1.19 h/day, respectively). (ii) Caring hours were not associated with differences in PA or SB (*p* > 0.05). (iii) Logistic regressions revealed very low adherence to PA guidelines among carers: 2% met the moderate-to-vigorous PA guideline, 26% met the muscle-strengthening guideline, and only 1% met the combined recommendations. Demographic and health variables did not explain adherence to these guidelines. This study found no significant differences in objectively measured PA and SB between informal carers and non-carers or caring hours. However, adherence to the UK CMOs’ PA guidelines among carers was extremely low. These findings provide the first objective benchmark of carers’ PA and SB patterns in Great Britain and highlight guideline adherence as a key area for future interventions. Future research should consider the wider context of caring in order to develop flexible, tailored interventions that can support carers in achieving an active lifestyle whilst managing responsibilities.

## 1. Introduction

Informal or unpaid carers play a vital but often overlooked role in health and social care systems. According to the Department of Health and Social Care, an informal or unpaid carer is “someone who provides unpaid help to a friend or family member needing support, perhaps due to illness, older age, disability, a mental health condition or an addiction” [1]. With increasing pressures on health and social care services across the world, more care is being delivered outside formal healthcare settings and within communities, often by family members or friends [2]. It is estimated that there are over 2 billion people providing unpaid care globally [3], although this figure is likely an underestimate, as many individuals do not recognise their caring role or self-identify as an informal carer [4].

The role of a carer has never been more important. As the world’s population ages and health services continue to face many challenges, carers have become essential to maintaining the health and independence of vulnerable individuals. For example, in the United Kingdom, informal carers are estimated to save the National Health Service (NHS) around £184 billion annually, a 29% increase from 2011 [5], yet they often do so at a high personal cost. Many carers report worsening mental and physical health, with over 80% stating that their caring responsibilities negatively affect their mental and physical wellbeing [6] and due to the demands of their role, they are unable to prioritise their own health and wellbeing [7]. Research has revealed that caregiving is associated with poorer mental health [8], including depression [9], and increases the risk of mortality due to the mental and emotional strain experienced [10]. When carers’ health declines, the repercussions extend beyond the individual, affecting the individuals they support and adding further strain to already stretched health and social care systems.

Caring responsibilities can also create unique and additional barriers to maintaining a healthy lifestyle, distinguishing carers from non-carers. Unlike individuals without caring roles, carers often face limited time, fatigue, financial constraints, and reduced opportunities to engage in physical activity (PA) due to the ongoing demands of providing care [11,12]. Feelings of guilt about leaving the care recipient or uncertainty about their safety can further restrict their participation in PA [11,12]. As a result, these characteristics may explain why carers may be less active than non-carers, but can also explain why many carers report engaging in less PA since taking on their caring role [13,14,15] despite the strong evidence that PA and reduced sedentary behaviour (SB) are beneficial for health [16,17,18]. The potential reduction or absence of PA is particularly concerning, given that carers’ wellbeing underpins not only their own health but also those who rely on them, and the wider economic systems that depend on them. From a health promotion and public health perspective, understanding carers’ PA and SB is critical for informing interventions and policies that support carers’ health, sustain their capacity to provide care, and reduce the pressures on health and social care systems. Generating robust population-level evidence is therefore essential for guiding effective support for informal carers.

Understanding the PA and SB patterns of informal carers is essential, not only to support this societal group directly but also to safeguard the sustainability of care provision. Previous research has examined PA among informal carers; however, much of the available evidence relies on self-reported measures or non-UK samples [19]. Epidemiological data describing carers’ PA and SB within the United Kingdom remain limited. For example, a systematic review conducted by Horne, Kentzer, Smith, Trott, and Vseteckova [11] that searched for the prevalence of PA of informal carers in the UK found no eligible primary studies that provided a single measure of PA prevalence in this target group, highlighting gaps in nationally representative evidence. Large-scale population datasets, particularly those with objective PA measures such as the 1970 British Cohort Study (BCS70), provide an opportunity to address this gap.

The BCS70 is an ongoing longitudinal study following individuals born in a single week across England, Scotland, and Wales [20]. The study follows the lives of these individuals from childhood (ages 5 and 10), adolescence (age 16), adulthood (ages 26, 30, 34, 38), and midlife (ages 42, 46, 51, and 56), typically through the use of interviews and surveys. Critically, participants are asked a question about the number of hours they spend during a week providing unpaid care, meaning the longitudinal dataset can be used to add to current knowledge on carer PA. Additionally, the Age 46 Survey (sweep 10/12 total planned sweeps), unlike any of the other age surveys of the BCS70, collected data from accelerometers enabling objective measurement of PA and SB patterns such as step count, activity time over the day, activity time for moderate to vigorous activities over the day, sitting time over the day, transitions from sitting to standing, and the number of sitting bouts over the day lasting more than 60 min. These accelerometer data have previously supported several studies reporting robust and significant findings, including evidence of the feasibility of capturing free-living SB in a large nationally representative cohort [21], statistically meaningful insights into how individuals accumulate stepping across postures and intensities [22], and significant associations between sitting time and grip strength [23]. Together, these strengthen the position of the BCS70 as a high-quality resource for examining patterns of PA and SB among carers in Great Britain.

Using the BCS70 dataset, we aimed to understand, at a national level, the PA and SB levels of informal carers, how these behaviours can be supported, and which groups of informal carers may benefit most from a tailored intervention. To guide this analysis, the following research questions were developed:

1. How do informal carers’ PA and SB patterns compare against non-carers’ PA and SB patterns?
2. How do the hours spent providing informal care influence PA and SB patterns?
3. What are the determinants of meeting the UK Chief Medical Officers’ PA guidelines among informal carers?

## 2. Materials and Methods

### 2.1. Design and Participants

The BCS70 Age 46 Survey data were analysed for the purpose of this research. The Age 46 Survey, comprising a home visit between 2016 and 2018 for data collection, meaning participants were between 46 and 48 at the time of data collection. The 1970 British Cohort Study has received full ethical approval from the NRES Committee South East Coast, Brighton and Sussex (Ref. 15/LO/1446).

### 2.2. Physical Activity and Sedentary Behaviour Measurement

In addition to surveys, interviews, and biomedical measures collected in the Age 46 Survey, this sweep also included the opportunity for participants to wear a thigh-mounted accelerometer, activPAL 3 micro device (PAL Technologies Ltd., Glasgow, UK) to gain an understanding of their movement behaviours.

The activPAL device is a triaxial accelerometer that uses thigh inclination and acceleration to estimate body posture (calculating 1 h periods as sitting/lying, standing, and walking), transitions between postures, stepping, and stepping speed. Sitting time (hours per day (h/d)) can also be calculated from these measurements taken. The thigh-mounted activPAL has been validated for the accurate assessment of sedentary time when compared with direct observation [24] and can be more precise in recording changes to sitting time than hip-worn monitors [25]. The activPAL devices were fitted by a nurse to the anterior midline of the participants’ right thigh, and participants were provided an accelerometer user guide with instructions to wear the device for 7 days during sleep, bathing, and all PA [26]. The accelerometers for this study used a previously described wear protocol that consisted of enabling 24 h wear, minimising data loss, and quality assurance [27]. Lastly, moderate to vigorous physical activity (MVPA) was derived using a step cadence threshold of ≥100 steps per minute [28] and valid waking wear time was calculated using a novel and previously validated algorithm that isolates valid waking wear time from sleep and prolonged non-wear [29].

The PA and SB data captured from the activPAL devices were used for the analyses to answer the first and second research questions of this study. In order to answer the third research question regarding the determinants of carers meeting the UK Chief Medical Officers’ (CMO) PA guidelines for adults age 19–64 years of accumulating; at least 150 min of moderate intensity activity; or 75 min of vigorous intensity activity or even shorter durations of very vigorous intensity activity, participating in muscle-strengthening activities at least 2 days per week [30], and meeting the overall recommendations (combined MVPA and muscle-strengthening guidelines), three new dichotomous variables (yes/no) had to be created based on the physical activity data captured by the activPAL devices.

The variable ‘mean activity time of moderate-to-vigorous intensity over the day’ (average of 7 days ‘B10AAMVPAH’) was used to determine if individuals met the UK CMOs’ MVPA minutes recommendation. As there was no differentiation between moderate- and vigorous-intensity PA captured from the activPAL data, a cutoff of 150 min (2.5 h) was used. If participants engaged in 150 min of more, they were scored ‘yes’ to meeting the MVPA aspect of the guidelines. It must be noted that the researcher is aware that this process may have excluded participants who recorded 75 min of vigorous activity or even less time of very vigorous activity, as the data provided by the activPAL did not differentiate the intensities of activities.

Self-reported muscle-strengthening activities reported in the BCS70 survey that reflected the definition of muscle-strengthening activities in the UK CMOs’ guidelines were used to indicate whether this element of the guidelines was met. These muscle-strengthening activities included: mowing the lawn, digging, shovelling, and chopping wood, exercising with weights, floor exercises, and martial arts/boxing/or wrestling. To meet the frequency of the guidelines, participants had to report taking part in the activity 2 to 3 times a week (scored 6), 4 to 5 times a week, (scored 7), or 6 times a week or more (scored 8). Participants who met this requirement were scored ‘yes’ to meeting this aspect of the guidelines.

Lastly, to understand if participants met both aspects of the CMOs’ guidelines an additional variable was created. If participants met both the recommended MVPA minutes and the muscle-strengthening guidelines, they were classed as meeting the overall guidelines and scored ‘yes’; participants who did not meet the overall guidelines were scored ‘no’.

### 2.3. Lifestyle and Health Measures

Demographic data and data relating to the participants’ lifestyles were collected through the main survey of the BCS70. These lifestyle and demographic variables included in analyses from the BCS70 dataset were chosen with consideration of Pearlin’s model of caregiver stress [31], incorporating the background of the informal carer, primary stressors, secondary role strains, secondary intra-psychic strains, with the outcome being PA and SB levels (Table 1).

Informal caregiving within the BCS70 cohort was operationalised using the survey variable ‘B10Q17G’, which asks participants to report the number of hours per week spent providing non-work-related care to elderly or disabled individuals. Participants reporting any hours of such care were classified as informal carers for the purposes of this analysis.

This operationalisation reflects the closest available approximation within the BCS70 to policy and conceptual definitions of informal caring, such as those used by the UK Department of Health and Social Care, which emphasise the provision of unpaid care to individuals with long-term illness, disability, or age-related needs. However, it should be noted that the survey-based measure does not capture contextual details such as the relationship to the care recipient, the nature of caring tasks, or the duration of care over time (e.g., how long they have been providing care to the care recipient).

Although the BCS70 includes a separate item assessing hours spent caring for pre-school children/babies, this question lacks sufficient contextual information to distinguish between routine parental responsibilities and providing unpaid care for a child with additional needs. As a result, this item was not used to identify potential informal carers to avoid misclassification. Furthermore, the BCS70 does not include measures to capture care provision for older children, which limits the identification of carers providing support to this group. These measurement constraints should be considered when interpreting the findings and their generalizability.

### 2.4. Statistical Analyses

Before statistical analysis was conducted in IBM SPSS Statistics (Version 29), the main survey data and the activPAL datasets (average and daily) were merged using RStudio (v.2025.05.1).

The distributions of the PA and SB variables were examined for normality and potential outliers, through normality testing using the Kolmogorov–Smirnov test and inspection of histograms. In addition to this, data were cleaned to remove ‘dress rehearsal’ data, conducted on a sub-sample of BCS70 participants to check the content and order of the interview, interview length, nurse protocols and instructions and design of the survey documents, participants who did not have 7 valid days of activPAL wear, participants with incorrect days of wear (e.g., 3 weekend days and 4 weekdays), to allow for accurate comparisons to be made between weekdays and weekends, and participants with missing data from any PA, SB, and/or lifestyle variables of interest.

To account for individuals with missing data, Chi-Square tests and Mann–Whitney U tests were conducted for categorical and continuous lifestyle and health variables. Any variable with a significant association between being included (no missing data) and excluded (missing data) in the analysis was controlled for during the analyses to answer our research questions. In addition, a logistic regression was conducted to account for carer and non-carer differences within our demographic data. Again, this was done to control for any statistically significant differences between these groups during the analyses.

To answer the research questions of this paper, analysis of covariance (ANCOVA) was used to compare PA and SB outcomes between carers and non-carers whilst controlling for significant differences between carers and non-carers and individuals with and without missing data. A series of ANCOVAs was also conducted to examine the association between hours of providing informal care and the PA and SB outcomes, controlling for significant differences between individuals with and without missing data.

Finally, to answer our third research question, a hierarchical logistic regression was conducted to understand the predictors of meeting the (1) MVPA minutes of the UK CMOs’ PA guidelines; (2) muscle strengthening aspect of the PA guidelines; and (3) overall UK CMOs’ PA guidelines for informal carers. Multicollinearity was checked across all independent variables (demographic variables) and our dependent variables (meeting the MVPA minutes of the UK CMO PA guidelines, meeting the muscle-strengthening guidelines, and meeting the combined guidelines). Once this assumption was met, the first step of the model included the relevant dependent variable, caring hours, and covariates (age and the 2015 index of multiple deprivation). All remaining demographic variables and the same covariates were included in the second step of the model. This approach aligns with Wong and Mason [32], who demonstrated the importance of modelling binary outcomes using systematically layered predictors. Our two-step modelling approach follows this logic by first isolating the effect of caring hours and then assessing the contributions of additional demographic characteristics.

## 3. Results

### 3.1. Data Cleaning

**Figure 1:**
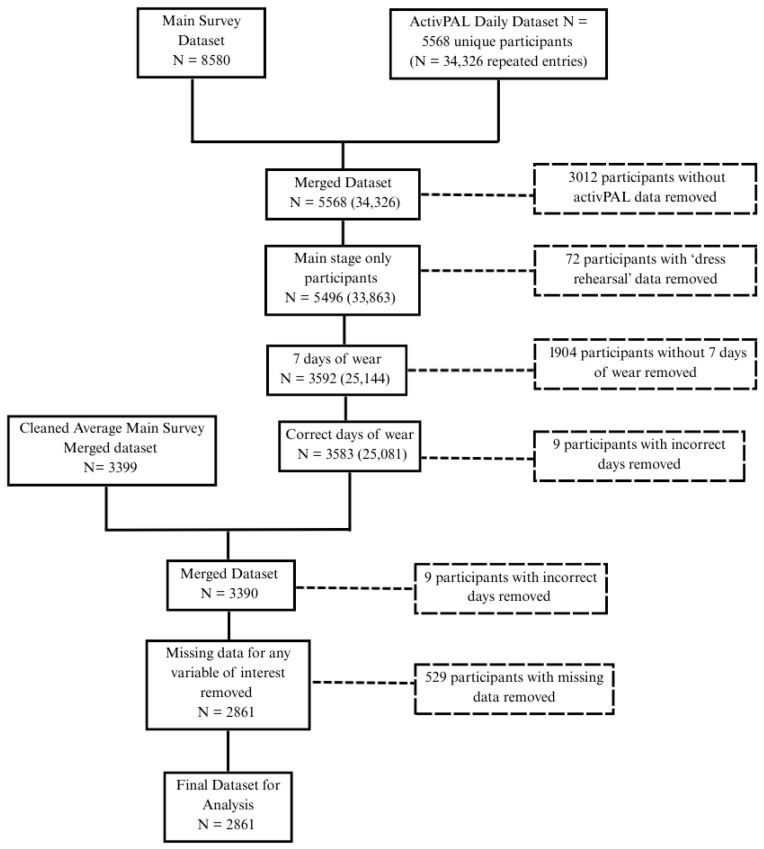
Data cleaning process.

Data were cleaned to remove participants with ‘dress rehearsal’ data (*n* = 72 participants removed), participants without 7 days of wear, as per the protocol, (*n* = 1094 participants removed), participants with incorrect days of wear (*n* = 9 participants removed), and participants with missing data from any variable of interest (*n* = 529 participants removed). Overall, a total of 2861 participants (*n* = 310 carers, *n* = 2551 non-carers) with valid activPAL and main survey responses were included in this analysis (Figure 1).

### 3.2. Normality Testing

PA and SB outcome data was not normally distributed, and therefore log transformations of the data were created. For PA and SB variables that did not include zero as an answer, the log transformation was Log_10_(x), and for PA and SB variables with a meaningful zero value, the log transformation was Log_10_(x + 1). The Kolmogorov–Smirnov values of the activity data after they were log transformed were still not normally distributed; however, skewness and kurtosis values were now within a more acceptable range [33] and closer to zero (mean skewness before = 0.828 vs. mean skewness after = 0.411). Therefore, transforming the data did successfully make the PA and SB variables comply with a more normal data distribution, allowing a parametric test to be conducted. For the purpose of easier interpretation, the PA and SB variables have been transformed back to their original scale.

### 3.3. Identifying Covariates

We took a data-driven approach [34,35] to identify variables that differed between informal carer and non-carer membership and should therefore be included as covariates in subsequent analyses. A binary logistic regression was conducted to assess the effect of demographic, health, and socioeconomic factors on the likelihood of being an informal carer.

Before this logistic regression was conducted, multicollinearity of the demographic, health and socioeconomic variables was assessed using a linear regression. Pearson’s correlations indicated strong correlations between the ‘SF-36 General health score’ and ‘general state of health’ (−0.816), and between ‘SF-36 emotional wellbeing score’ and ‘Warwick–Edinburgh Mental Wellbeing Scale’ (0.756). To minimise collinearity, the ‘SF-36 general health score’ and ‘SF-36 emotional wellbeing score’ variables were excluded from subsequent analyses.

The final logistic regression model was statistically significant when compared to the null model (χ^2^(25) = 155.750, *p* < 0.001), explained 10.7% of the variance in being an informal carer, and correctly classified 89.4% of all participants. Sex, current economic activity status, and BMI classification had statistically significant differences between carers and non-carers; being male (Exp(B) 0.595), in full-time or part-time employment (Exp(B) 0.186 and 0.298 respectively) and being overweight or obese (Exp(B) 0.465 and 0.426 respectively) had lesser odds of being a carer and greater odds of being a non-carer.

In addition to covariates of informal carer membership, covariates to control for the impact of data exclusion (or missing data) were analysed. Chi-square and Mann–Whitney U tests were conducted to assess demographic differences between participants who were included and excluded from the analysis. Variables with statistically significant differences were age (*p* < 0.001) and the 2015 index of multiple deprivation (χ^2^(10) = 48.480, *p* < 0.001). Post hoc analyses revealed that participants were older in the excluded group; individuals from the most deprived and least deprived deciles were less likely to be excluded from the sample than expected; and individuals from the mid-upper decile (rank 8) were more likely to be excluded from the sample than expected.

Taken together, variables that needed to be controlled for were sex, current economic activity status, BMI classification, age, and the 2015 index of multiple deprivation.

### 3.4. Carer Participant Characteristics

From a total population of 310 carers who were included in this study, 70% were female (*n* = 217) and 30% were male (*n* = 93). As this sample of carers was all born in the same week in 1970, they should be the same age; however, because data collection was between 2016 and 2018, their ages differed slightly. The mean age of the sample was 46.79 (SD = 0.685).

Of the 310 carers that agreed to wear the activPAL device, 50 (16%) provided <1 h of care per week, 79 (25%) provided between 1 and 3 h of care per week, 44 (14%) provided between 3 and 6 h of care per week, 29 (9%) provided 6–10 h of care per week, 14 (4%) provided 10–15 h of care per week, and 94 (30%) provided 15 or more hours of care per week.

### 3.5. Research Question 1

Analyses of covariance (ANCOVAs) were conducted to compare carers and non-carers across the various PA and SB outcomes (*n* = 18 separate ANCOVAs), controlling for relevant covariates (sex, current economic activity status, BMI classification, age, and 2015 index of multiple deprivation). All dependent variables were log-transformed to address violations of normality.

Assumptions of linearity of the dependent variables and continuous covariates, and homogeneity of regression slopes were met. Levene’s test indicated potential violations of homogeneity of variances for variables: ‘mean activity time over the day (moderate-to-vigorous)’, ‘weekend sitting time over the day’, and ‘weekend number of sitting bouts over the day lasting 60+ minutes’. However, the test is known for over-sensitivity in large samples or data which deviate from normality [36]. Visual inspection of histograms, boxplots, and means and standard deviations were very similar, meaning the ANCOVAs were still conducted.

After adjusting for our covariates, we found no statistically significant differences between carers and non-carers across all PA and SB outcomes, including 7-day averages, weekday averages, and weekend averages (all *p*-values > 0.05, with a negligible effect size) (Table 2).

In conclusion, although there are some small differences in the means of carers’ and non-carers’ PA and SB outcomes, these differences are not statistically significant. Therefore, being an informal carer was not associated with significant changes in PA and SB outcomes after accounting for covariates.

### 3.6. Research Question 2

Analyses of covariance (ANCOVAs) were conducted to examine the association between hours of providing informal care and various PA and SB outcomes (N = 18 separate ANCOVAs), controlling for relevant covariates (age and 2015 index of multiple deprivation). All dependent variables were log-transformed to address violations of normality.

Assumptions of linearity between the dependent variables and the continuous covariate, and the homogeneity of regression slopes were met. Levene’s test indicated potential violations of homogeneity for ‘weekend daily step count’ and ‘weekend activity time for moderate to vigorous activity’. However, visual inspection of histograms and boxplots showed that means and standard deviations were similar, and so the relevant ANCOVAs were still conducted.

Across all outcomes—including mean daily step count, weekday and weekend step count, total and moderate-to-vigorous activity time, sitting time, number of sit-to-stand transitions, and prolonged sitting bouts—there were no statistically significant differences between the number of hours spent providing care (all *p*-values > 0.05, with a minimal effect size). For example, ANCOVA revealed no significant differences in mean daily step count by caring hours, F (5, 302) = 0.934, *p* = 0.459, or in mean sitting time, F (5, 302) = 1.232, *p* = 0.294. Overall, caring hours were not associated with meaningful differences in PA or SB in this sample of carers (Table 3).

### 3.7. Research Question 3

To address our third research question, a hierarchical logistic regression was performed to identify predictors of informal carers meeting the UK CMOs’ PA guidelines of 150 min of MVPA as recorded using activPAL, muscle-strengthening activities at least 2 days per week through self-reported measures, and the overall guidelines (these elements combined). Prior to conducting the regression, multicollinearity among all independent variables (lifestyle and health measures) and the dependent variable, (1) meeting the MVPA guidelines, (2) meeting the strength guidelines, (3) meeting the overall guidelines, was assessed, with no significant multicollinearity being found.

**Figure 2:**
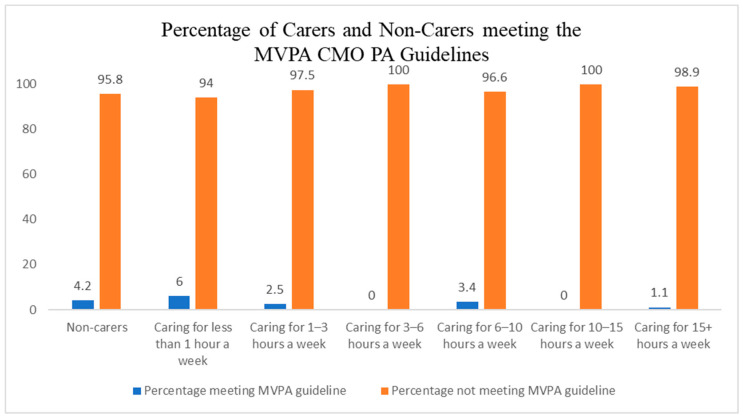
Percentage of carers and non-carers meeting the moderate-to-vigorous physical activity aspect of the UK chief medical officers’ physical activity guidelines.

**Figure 3:**
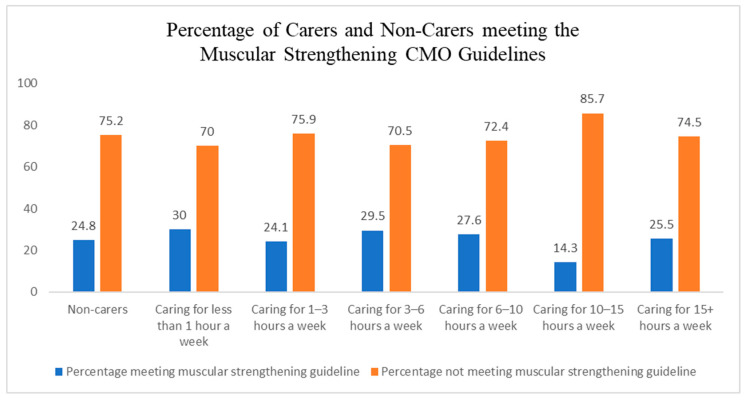
Percentage of carers and non-carers meeting the muscle strengthening aspect of the UK chief medical officers’ physical activity guidelines.

**Figure 4:**
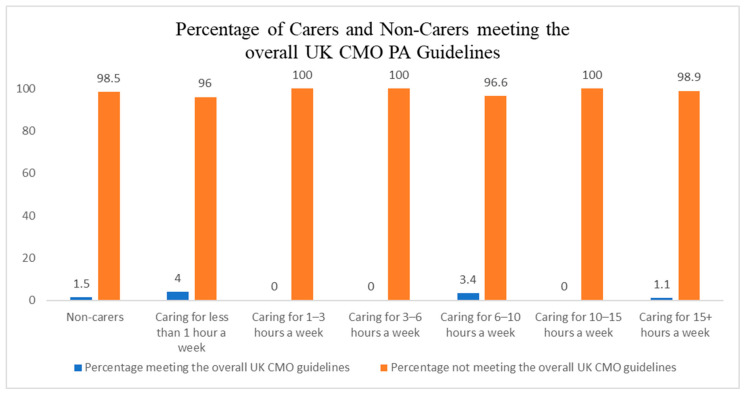
Percentage of carers and non-carers meeting the UK chief medical officers’ physical activity guidelines.

Out of the total sample of 310 informal carers, only 7 (2%) achieved at least 150 min of moderate PA in the week (Figure 2), 81 (26%) met the muscle-strengthening guideline (Figure 3), and 4 (1%) met the overall guidelines (Figure 4).

#### 3.7.1. MVPA

The first model of logistic regression included caring hours and covariates age and the 2015 index of multiple deprivation as predictors. The model was not statistically significant χ^2^(15) = 16.972, *p* = 0.321, meaning this combination of variables did not significantly predict whether carers met the guidelines of 150 min moderate PA. This model accounted for 27.4% of the variance (Nagelkerke R^2^ = 0.274) and correctly classified 97.7% of cases.

In the second model of the regression, additional demographic variables (sex, current economic activity, general health score, BMI classification, Warwick–Edinburgh mental wellbeing scale, and total malaise score) were included in the model along with the same covariates (age and 2015 index of multiple deprivation) and caring hours. This expanded model also did not result in statistical significance, χ^2^(30) = 37.893, *p* = 0.153. However, it explained a greater proportion of the variance (Nagelkerke R^2^ = 0.593) and correctly classified 97.4% of cases.

Overall, although the second model demonstrated an improved fit and explained greater variance, neither of the steps from this logistic regression produced a statistically significant model in predicting whether informal carers met the 150 min of the UK CMOs’ PA guidelines.

#### 3.7.2. Muscle Strengthening

The first model of the logistic regression included caring hours and covariates age and the 2015 index of multiple deprivation as predictors. The model was not statistically significant χ^2^(15) = 13.68, *p* = 0.550, meaning this combination of variables did not significantly predict whether carers met the muscular strengthening guidelines. This model accounted for 6.3% of the variance (Nagelkerke R^2^ = 0.063) and correctly classified 73.9% of cases.

In the second model of the regression, additional demographic variables (sex, current economic activity, general health score, BMI classification, Warwick–Edinburgh Mental Wellbeing Scale, and total malaise score) were included in the model along with the same covariates (age and 2015 index of multiple deprivation) and caring hours. This expanded model also did not result in statistical significance, χ^2^(30) = 27.48, *p* = 0.598. However, it explained a greater proportion of the variance (Nagelkerke R^2^ = 0.124) and correctly classified 73.9% of cases.

In conclusion, neither model significantly predicted adherence to the muscular strengthening aspect of the UK CMOs’ guidelines. However, the inclusion of additional demographic variables modestly improved the explained variance.

#### 3.7.3. Overall Guidelines

Out of the 310 informal carers in the sample, only 4 (1%) participants met the combined PA guidelines for MVPA minutes and muscle-strengthening activities. Due to the small sample of carers who met the guidelines and the greater number of predictor variables, a logistic regression was not conducted to avoid drawing conclusions from analyses with very small sample sizes. Instead, comparisons between carers who met the guidelines and carers who did not meet the guidelines are presented descriptively (Table 4).

Caring hours, sex, age, socioeconomic status, and employment showed little variation in relation to guideline adherence, with carers meeting the guidelines found across different categories. Carers who met the guidelines were aged 46–47, in full-time employment, part-time employment, or not working, and were distributed across the most deprived, second least deprived and middle ranks of the socioeconomic deciles.

Self-reported general state of health revealed some differentiation, as only those who rated their health as good, very good, or excellent met the guidelines, whilst those with fair or poor health did not. BMI and psychological health indicators also highlighted a more distinct pattern between those meeting and not meeting the overall MVPA and strength guidelines. Three carers of healthy weight and one who was obese met the guidelines, whereas those underweight, overweight, or morbidly obese did not. All four carers who met the guidelines were grouped in the low malaise score group and had a higher Warwick–Edinburgh mental wellbeing scale score.

In conclusion, guideline adherence was strikingly low amongst this sample of informal carers. While sociodemographic variables such as sex, age, caring hours, and socioeconomic status did not appear to strongly influence adherence, indicators of general health, BMI, and psychological wellbeing showed some association with meeting the combined overall guidelines.

Finally, post hoc analysis revealed that non-carers were also not meeting the recommended PA levels set by the UK CMO (Table 5).

## 4. Discussion

### 4.1. Overall Findings

This secondary data analysis of the BCS70 revealed that after accounting for covariates, there were no statistically significant differences between carers and non-carers across all PA and SB outcomes, and that there were no statistically significant differences between the number of hours spent providing informal care and PA and SB outcomes. Lastly, this analysis revealed that there is a strikingly low proportion of carers (1%, *n* = 4) meeting the UK CMOs’ PA guidelines of 150 min of moderate physical activity and muscle-strengthening activities at least twice a week, with no demographic or health variables able to statistically explain adherence or lack of adherence to these guidelines.

### 4.2. Carer vs. Non-Carer Physical Activity and Sedentary Behaviour

The present study directly addressed the evidence gap highlighted in the introduction, noting that UK- or GB-based epidemiological data on carers’ PA and SB were noted to be scarce. Using large-scale, objectively measured data from the BCS70, we were able to compare movement behaviours between carers and non-carers in Great Britain. Our analyses revealed no significant differences in objectively measured PA and SB between carers and non-carers in Great Britain across all outcomes, including daily step count, activity time, moderate-to-vigorous activity time, and sitting time. Importantly, these null findings are themselves important. The use of large-scale, objectively measured activPAL data strengthens the confidence that the absence of differences between carers and non-carers is unlikely to reflect measurement bias or insufficient statistical power.

These findings are consistent with those of Marquez, Bustamante, Kozey Keadle, Kraemer, and Carrion [37], who also reported no significant difference in accelerometer-derived PA between older caregivers and non-caregivers (all *p*-values > 0.11). Similarly, a systematic review by Lindsay, Vseteckova, Horne, Smith, Trott, De Lappe, Soysal, Pizzol and Kentzer [19] identified nine studies (one of which was Marquez et al. [36]), five of which used data from population-based studies, that revealed no significant differences between carers and non-carers PA levels. Together, these findings suggest that caring status alone may not be a sufficient determinant of movement behaviours. This challenges assumptions that informal caring is inherently associated with lower PA and higher SB, particularly when examined using objective measures and accounting for key sociodemographic factors.

An alternative interpretation of these findings is that broader structural constraints may influence PA and SB in carers and non-carers alike. Factors related to societal constraints and the socioecological model, such as physical environments, local authority policies, social support, and societal norms, may have a greater influence on movement behaviours than caring status. Therefore, in relation to this study, carers and non-carers may experience comparable constraints on opportunities for PA, resulting in similar overall PA and SB levels despite differences in caring responsibilities.

However, work by Lindsay, Vseteckova, Horne, Smith, Trott, De Lappe, Soysal, Pizzol and Kentzer [19] also reported contrasting findings between carers and non-carers. Findings from several studies included in their systematic review, such as analyses of national survey data from 38 low and middle income countries, a population based study in Hong Kong, and data from 50 US states, reported that carers engaged in more overall PA than non-carers and patients, and carers aged 18 to 64 had a lower risk of physical inactivity compared to non-carers [38,39,40]. Meanwhile, six studies from this systematic review found that non-carers engaged in more PA than carers [37,41,42,43,44,45,46,47,48].

When comparing the PA and SB levels of informal carers in this sample with those reported in other objective caregiving studies, carers in this cohort appear to be more active and less sedentary overall. For example, family carers of individuals with multiple sclerosis in Fakolade, Finlayson, Parsons and Latimer-Cheung [49] averaged 6160 steps per day and only 21.4 min of MVPA, while a work by Carpenter, Miller, Sui and West [50] reported that carers of people living with Alzheimer’s disease spent an average of 769.4 min (12.8 h) engaged in SB. In contrast, carers in the current study achieved over 9000 steps per day on average, roughly 1 h of MVPA during weekdays and weekends, and 9 h of sitting time, indicating a more active lifestyle overall.

These discrepancies likely reflect variation in care intensity, role demands, and population characteristics rather than inconsistency in the evidence base. Much of the existing literature has focused on older, higher-burden caregivers, often providing full-time care for individuals with chronic or disabling conditions, where time constraints, fatigue, and psychological strains may substantially constrain opportunities for PA. In contrast, the present study population-based sample did not include older carers and lacked detailed contextual information about their caring role, responsibilities, and the care recipient. This broader carer profile may help to explain both the relatively higher PA levels observed among carers and the absence of significant differences between carers and non-carers in this study.

Overall, these findings suggest that informal caring does not inevitably lead to reduced PA or increased SB, and that population-level movement behaviours may be shaped more strongly by shared structural and socioecological factors than by caring status alone.

### 4.3. Caring Hours

Findings from this analysis revealed that there were no statistically significant differences in PA and SB levels across the number of hours spent providing care. One possible explanation for the lack of significant differences between caring-hour groups was the way in which caring hours were categorised in the BCS70 dataset. The highest group was defined as those providing 15 or more hours of care per week, which may not adequately capture differences between part-time and full-time carers. Previous research has indicated that higher caregiving intensity can affect PA behaviours. Hajek, Bock, and König [51] aimed to examine the association of informal caregiving on BMI and frequency of sporting activities using cross-sectional data from the German Ageing Survey. Findings from Hajek, Bock, and König [51] revealed that the frequency of sporting activities significantly decreased with caregiving time, with each additional hour spent providing care being associated with a lower frequency of sporting activities. Therefore, the grouping of the data within the BCS70 may have hidden potential differences between individuals caring for fewer hours per week (e.g., <1) and those providing substantially greater hours of care, such as 35 h (full-time) or even more hours per week.

Additionally, it is possible that the relationship between providing informal care and PA may be influenced by other factors not captured by caring hours alone. For example, the type and intensity of the caring role, the care recipient’s level of dependency, the carers’ attitude towards physical activity (in relation to the transtheoretical model), and the support the carer receives (e.g., respite) could impact their ability and motivation to engage in PA and SB. Psychological and emotional strain associated with the caring role, such as increased stress and depressive symptoms have been found to be associated with a reduced frequency of PA [44]. Therefore, suggesting the context of the care provided may influence PA levels alongside psychological and emotional factors.

### 4.4. Meeting PA Guidelines

This study found that adherence to the UK CMOs’ PA guidelines among informal carers was extremely low. Only 2% met the MVPA recommendation of at least 150 min per week; 26% met the muscle-strengthening recommendations; and just 1% achieved both components of the guidelines. Logistic regression analyses indicated that neither caring hours nor demographic or health-related factors significantly predicted adherence to the guidelines. These findings indicate that engagement with recommended PA levels among carers in this sample was minimal and not readily explained by individual, sociodemographic, or health characteristics.

The exceptionally low levels of guideline adherence also warrant reflection on how responsibility for PA and health is conceptualised in the context of caring. Much of the PA guidance implicitly adopts an individual model of resilience, in which carers are expected to maintain their own health by finding time, motivation, and resources to be physically active despite substantial caring responsibilities. When interpreted with this in mind, low adherence risks being framed as a failure of individual behaviour or motivation. However, this interpretation overlooks the structural realities of caring and risks placing responsibility on carers for outcomes that are shaped by broader system-level constraints, over which they often have limited control.

Compared with existing literature, these findings suggest far lower levels of activity among carers than previously reported. Lindsay, Vseteckova, Horne, Smith, Trott, De Lappe, Soysal, Pizzol and Kentzer [19] found that between 16 and 84% of carers in low quality studies, and 29.9 to <99% in high quality studies, did not meet the WHO PA guidelines, of at least 150–300 min of moderate intensity aerobic activity or 75 min of vigorous intensity activity per week, and muscle-strengthening activities two times per week [52]. In contrast, only 1% of carers in this study sample met the overall UK CMO guidelines, of at least 150 min of moderate intensity activity and muscle-strengthening activities twice per week, indicating a substantially greater degree of inactivity. This discrepancy is likely influenced by methodological differences. Much of the prior literature relied on self-reported PA measures such as the IPAQ, PASE, and RAP, which are known to overestimate PA relative to objective measures [53,54,55]. The use of objective activPAL data and a strict MVPA threshold in the current study likely provides a more conservative but accurate estimate of guideline adherence, suggesting that previous studies may have overestimated carers’ engagement in PA.

Post hoc analysis revealed that non-carers were also largely inactive; however, non-carers were almost twice as likely to meet the MVPA minutes compared to carers (4.2% vs. 2.3%, respectively). Although overall adherence remained low in both groups, this disparity suggests that carers may face additional constraints to engaging in sufficient PA. When benchmarked against national self-report data, inactivity among carers appears particularly concerning. For example, 33% of adults aged 45–54 in Scotland met both the MVPA and strength guidelines [56], while 57% in Northern Ireland [57] and 35% in Wales [58] report regular participation in PA, and 22% of adults in England are classified as active [59]. Although these health reports did not specify activity levels of specific populations, are based on self-reported data, and are not specific to the caring population, they suggest that carers may be among the least active groups in the population and face unique challenges to engaging in regular PA.

Several factors may help explain the strikingly low adherence observed. However, these should be understood within a broader ecological context rather than as individual-level shortcomings. Caring responsibilities are often embedded within systems that limit access to time, flexibility, respite, and supportive services, which can therefore limit opportunities for structured or leisure-time PA. Previous research has identified barriers such as fatigue, health problems, lack of motivation, reluctance to leave the care recipient, limited flexibility in daily routines, and emotional burden as key obstacles to PA participation among carers [11]. Importantly, these barriers are not merely personal challenges but reflect the wider organisational and social contexts in which caring takes place. Many of these constraints were not captured by the logistic regression models used in the present study, which focused on individual-level predictors.

From this perspective, an ecological model of resilience offers a more appropriate framework for interpreting the findings. Resilience is not something carers are expected to generate independently, but rather something that is enabled or undermined by the environment, services, and policies around them. For carers, opportunities to engage in and sustain PA levels without attending to these enabling conditions effectively shift responsibility away from systems onto individuals who often have limited control over their circumstances.

Descriptive comparisons within the sample indicated that carers who met the PA guidelines were more likely to report good or better self-rated health, healthier BMI classifications, and higher wellbeing scores. This pattern aligns with previous research demonstrating strong links between PA, physical health, and mental wellbeing [60,61]. However, these associations should not be interpreted as evidence that improved health alone enables guideline adherence; rather, they likely reflect the cumulative advantage of having greater capacity and fewer constraints within an already demanding caring context.

Overall, the findings indicate that informal carers are at particularly high risk of physical inactivity, with adherence to the UK CMO PA guidelines far below the national averages. Rather than reflecting individual failure, these patterns point to structural and systemic barriers that limit carers’ ability to prioritise their own physical health. Addressing inactivity among carers, therefore, represents a key public health and health promotion challenge. Rather than focusing solely on individual behaviour change, effective responses require system-level strategies, including access to respite, flexible support services, supportive community environments, and policies that recognise and protect carers’ physical health alongside their caring responsibilities.

### 4.5. Strengths

This study had several notable strengths. A key strength of this study is the use of a validated and objective measurement tool to capture PA and SB patterns of informal carers through the use of the activPAL device, which allows reliable and accurate conclusions to be drawn about their activity levels and reduces the bias often associated with self-reported measures. To our knowledge, this is the first study to objectively evaluate the PA and SB levels of informal carers in Great Britain, offering novel insights into this important but understudied population group. Finally, this study analysed a relatively large sample of self-declared informal carers, substantially exceeding the number that could feasibly be recruited through primary data collection methods.

### 4.6. Limitations

Several limitations should be considered when interpreting these findings. First, because all participants were roughly 46 years old, the nature of the original BCS70 study’s aim restricts the generalizability of carers to other age groups, whose PA patterns and care duties may differ substantially. Additionally, the lack of carers supporting children or young people limits the generalizability of our findings to the caring population, which may face different time demands, stressors, and PA constraints.

This study lacked detailed contextual information about the caring role, including the type or intensity of care provided and the nature of PA undertaken whilst providing care. This limits the ability to directly compare the findings with previous research reporting on the prevalence of PA within informal carers. The statistical power for research question 3 was limited, with few carers meeting the guidelines and being included in the analysis, which may have reduced the precision and reliability of these findings. Lastly, the data was collected almost a decade ago, and PA behaviours may have evolved since then due to technological developments and commercialisation of health and fitness tracking, as well as broader societal changes following the COVID-19 pandemic. These factors should be considered when interpreting the relevance of the findings to current populations of informal carers.

### 4.7. Future Work

Future work should employ longitudinal designs to examine how informal carers’ PA and SB patterns change over time, including during key transitions into and out of caring roles. The use of objective measurement tools, such as accelerometry, would strengthen the accuracy of PA and SB assessment and reduce reliance on self-report.

Future studies should also aim to include more diverse carer populations, capturing variation in age, care intensity, type of care provided, and care recipient (e.g., caring for adults versus children). This would improve the generalizability of the findings and enable a deeper analysis of relationships between informal caring and movement behaviours.

To better understand the contextual factors shaping carers’ PA and SB, mixed-methods approaches are recommended. Qualitative components could explore the carers’ responsibilities and the impact caring duties have on carers’ PA and SB patterns, which could inform the design of a feasible and acceptable intervention to support PA and SB.

## 5. Conclusions

This secondary data analysis aimed to understand PA and SB patterns of informal carers within Great Britain. More specifically, how carers’ PA and SB levels compared with non-carers, if the hours of care provided influenced activity levels, and if there were any predictors for carers meeting the UK CMOs’ PA guidelines.

Using data from the BCS70, the findings suggested that, after accounting for relevant covariates, there were no statistically significant differences between carers and non-carers across all objectively measured PA and SB outcomes, and that there were no statistically significant differences between the number of hours spent providing informal care and objectively measured PA and SB outcomes. Lastly, this analysis revealed a strikingly low proportion of carers meeting the UK CMOs’ PA guidelines of 150 min of MVPA and muscle-strengthening activities at least twice a week, with no demographic or health variables statistically explaining adherence to or non-adherence to these guidelines.

Taken together, these findings indicate that while informal caring status and caring hours may not be associated with higher or lower PA and SB, carers may still face challenges in achieving recommended levels of PA. Further research is needed to explore contextual, behavioural, and structural factors related to caring that may influence PA engagement and to inform the development of tailored interventions.

## Figures and Tables

**Table 1:** Lifestyle and health variables.

Demographic and Lifestyle Variable Name	Type	Levels
Sex	Categorical	Male
		Female
Age	Continuous	N/A
Current economic activity status [recoded]	Categorical	Full-time employment
		Part-time employment
		Non-work
		Sickness/disability
		Other
BMI classification (based on nurse measurement)	Categorical	Underweight (below 18.5 kg/m^2^)
		Healthy weight (18.5–24.9 kg/m^2^)
		Overweight (25–29.9 kg/m^2^)
		Obese (30–39.9 kg/m^2^)
		Morbidly obese (over 40 kg/m^2^)
Warwick–Edinburgh mental wellbeing scale	Continuous	N/A
Total malaise score	Categorical	Low malaise (0–3)
		High malaise (4+)
2015 index of multiple deprivation rank decile	Categorical	Most deprived
		Least deprived
Number of hours spent caring for elderly/disabled people (non-work)	Categorical	None
		Less than 1 h per week
		1–3 h per week
		3–6 h per week
		6–10 h per week
		10–15 h per week
		15+ hours per week

**Table 2:** Physical activity and sedentary behaviour outcomes across carers and non-carers.

Variable (DV)	ANCOVA Result ^a^	Partial Eta Square	Mean (SD) ^b^	
			Carer	Non-Carer
Mean daily step count (steps/day)	F (1, 2854) = 0.156, *p* = 0.693	0.000	9316.06 (3440.24)	9554.11 (3466.01)
Weekday daily step count (steps/day)	F (1, 2854) = 0.001, *p* = 0.973	0.000	9498.65 (3685.31)	9577.54 (3722.21)
Weekend daily step count (steps/day)	F (1, 2854) = 0.752, *p* = 0.386	0.000	8862.86 (4002.73)	9498.53 (4401.40)
Mean activity time over day (h/day)	F (1, 2854) = 0.187, *p* = 0.665	0.000	2.00 (0.68)	2.00 (0.67)
Weekday activity time over day (h/day)	F (1, 2854) = 0.613, *p* = 0.434	0.000	2.02 (0.74)	1.99 (0.73)
Weekend activity time over day (h/day)	F (1, 2854) = 0.087, *p* = 0.768	0.000	1.93 (0.77)	2.02 (0.81)
Mean activity time (mod-vig) over day (h/day)	F (1, 2854) = 1.643, *p* = 0.200	0.001	0.82 (0.40)	0.86 (0.41)
Weekday activity time (mod-vig) over day (h/day)	F (1, 2854) = 0.124, *p* = 0.725	0.000	1.17 (0.47)	1.19 (0.48)
Weekend activity time (mod-vig) over day (h/day)	F (1, 2854) = 1.646, *p* = 0.200	0.001	1.09 (0.54)	1.19 (0.58)
Mean sitting time over day (h/day)	F (1, 2854) = 0.040, *p* = 0.842	0.000	9.15 (1.94)	9.25 (1.83)
Weekday sitting time over day (h/day)	F (1, 2854) = 0.002, *p* = 0.968	0.000	9.26 (2.12)	9.42 (2.11)
Weekend sitting time over day (h/day)	F (1, 2854) = 0.373, *p* = 0.541	0.000	8.87 (2.36)	8.83 (2.08)
Mean number of transitions sit-stand	F (1, 2854) = 0.000, *p* = 0.992	0.000	55.38 (15.13)	55.33 (15.33)
Weekday number of transitions sit-stand	F (1, 2854) = 0.118, *p* = 0.731	0.000	57.04 (15.77)	57.52 (17.02)
Weekend number of transitions sit-stand	F (1, 2854) = 1.298, *p* = 0.255	0.000	52.70 (17.33)	51.33 (16.07)
Mean number of sitting bouts over day lasting 60+ min	F (1, 2854) = 0.318, *p* = 0.573	0.000	1.47 (0.85)	1.50 (0.82)
Weekday number of sitting bouts over day lasting 60+ min	F (1, 2854) = 0.007, *p* = 0.931	0.000	1.44 (0.92)	1.47 (0.93)
Weekend number of sitting bouts over day lasting 60+ min	F (1, 2854) = 2.140, *p* = 0.144	0.001	1.56 (1.17)	1.58 (1.06)

^a^ ANCOVA performed on log-transformed PA and SB data. ^b^ Descriptives are based on untransformed data for interpretability.

**Table 3:** Physical activity and sedentary behaviour outcomes across caring hours.

Variable (DV)	ANCOVA Result ^a^	Partial Eta Square	Mean (SD) ^b^					
			<1 h/Week	1–3 h /Week	3–6 h/Week	6–10 h/Week	10–15 h/Week	15+ h/Week
Mean daily step count (steps/day)	F (5, 302) = 0.934, *p* = 0.459	0.015	9771.92 (3964.84)	9251.42 (3607.33)	9360.36 (3116.45)	10,228.97 (2826.62)	8223.29 (3273.61)	8988.30 (3326.91)
Weekday daily step count (steps/day)	F (5, 302) = 0.960, *p* = 0.442	0.016	9673.87 (3936.98)	9534.43 (4034.27)	9679.28 (3609.88)	10,264.22 (3133.70)	7966.17 (3423.18)	9282.88 (3474.57)
Weekend daily step count (steps/day)	F (5, 302) = 1.212, *p* = 0.303	0.020	10,020.30 (4866.84)	8547.11 (3535.80)	8566.50 (3274.57)	10,143.66 (4276.41)	8869.79 (4034.17)	8255.12 (3974.74)
Mean activity time over day (h/day)	F (5, 302) = 0.487, *p* = 0.786	0.008	2.07 (0.79)	1.97 (0.74)	1.98 (0.60)	2.12 (0.51)	1.84 (0.68)	1.98 (0.66)
Weekday activity time over day (h/day)	F (5, 302) = 0.622, *p* = 0.683	0.010	2.04 (0.81)	2.00 (0.81)	2.03 (0.72)	2.12 (0.56)	1.78 (0.72)	2.04 (0.71)
Weekend activity time over day (h/day)	F (5, 302) = 0.989, *p* = 0.425	0.016	2.14 (0.90)	1.90 (0.74)	1.84 (0.61)	2.13 (0.80)	1.98 (0.79)	1.82 (0.78)
Mean activity time (mod-vig) over day (h/day)	F (5, 302) = 1.613, *p* = 0.156	0.026	0.84 (0.45)	0.85 (0.39)	0.83 (0.40)	0.96 (0.36)	0.66 (0.36)	0.76 (0.39)
Weekday activity time (mod-vig) over day (h/day)	F (5, 302) = 1.191, *p* = 0.314	0.019	1.22 (0.51)	1.17 (0.50)	1.21 (0.46)	1.27 (0.41)	0.96 (0.43)	1.13 (0.45)
Weekend activity time (mod-vig) over day (h/day)	F (5, 302) = 1.495, *p* = 0.191	0.024	1.27 (0.69)	1.03 (0.44)	1.08 (0.43)	1.27 (0.61)	1.12 (0.59)	1.00 (0.52)
Mean sitting time over day (h/day)	F (5, 302) = 1.232, *p* = 0.294	0.020	8.83 (2.05)	8.96 (1.94)	9.69 (1.70)	9.37 (1.81)	9.15 (2.13)	9.16 (2.00)
Weekday sitting time over day (h/day)	F (5, 302) = 0.885, *p* = 0.491	0.014	9.07 (2.30)	9.06 (2.06)	9.76 (1.91)	9.49 (1.94)	9.28 (2.43)	9.22 (2.16)
Weekend sitting time over day (h/day)	F (5, 302) = 1.445, *p* = 0.208	0.023	8.23 (2.23)	8.72 (2.54)	9.50 (2.18)	9.07 (2.07)	8.84 (2.69)	9.00 (2.34)
Mean number of transitions sit-stand	F (5, 302) = 0.276, *p* = 0.926	0.005	54.86 (15.54)	56.70 (14.83)	54.84 (14.68)	55.03 (11.68)	58.36 (24.05)	54.46 (14.93)
Weekday number of transitions sit-stand	F (5, 302) = 0.334, *p* = 0.892	0.006	56.60 (15.96)	58.36 (15.29)	55.46 (14.84)	56.52 (11.41)	60.81 (23.80)	56.51 (16.44)
Weekend number of transitions sit-stand	F (5, 302) = 0.508, *p* = 0.770	0.008	51.63 (19.65)	54.04 (17.07)	54.88 (17.97)	52.95 (14.98)	53.50 (26.50)	50.95 (15.07)
Mean number of sitting bouts over day lasting 60+ mins	F (5, 302) = 0.602, *p* = 0.699	0.010	1.37 (0.68)	1.40 (0.87)	1.63 (0.87)	1.51 (0.90)	1.41 (0.80)	1.52 (0.92)
Weekday number of sitting bouts over day lasting 60+ mins	F (5, 302) = 0.839, *p* = 0.523	0.014	1.35 (0.83)	1.34 (0.91)	1.65 (0.97)	1.51 (0.91)	1.29 (0.80)	1.47 (0.95)
Weekend number of sitting bouts over day lasting 60+ mins	F (5, 302) = 0.325, *p* = 0.898	0.005	1.42 (1.02)	1.54 (1.23)	1.60 (1.03)	1.50 (1.32)	1.71 (1.28)	1.62 (1.21)

^a^ ANCOVA performed on log-transformed PA and SB data. ^b^ Descriptives are based on untransformed data for interpretability.

**Table 4:** Demographic characteristic comparison of carers who met and did not meet the UK chief medical officers’ physical activity guidelines.

Characteristic	Category	Met Guideline (*n*)	Not Met Guideline (*n*)	% Met
Caring hours	<1 h week	2	48	4.0
	1–3 h week	0	79	0.0
	3–6 h week	0	44	0.0
	6–10 h week	1	28	3.4
	10–15 h week	0	14	0.0
	15+ h week	1	93	1.1
Sex	Male	1	92	1.1
	Female	3	214	1.4
Age	46	1	110	0.9
	47	3	149	2.0
	48	0	47	0.0
	Mean	46.75	46.79	-
2015 index of multiple deprivation	1 most deprived	1	23	4.2
	2	0	19	0.0
	3	0	30	0.0
	4	0	24	0.0
	5	1	30	3.2
	6	0	42	0.0
	7	0	28	0.0
	8	0	36	0.0
	9	2	31	6.1
	10 least deprived	0	43	0.0
Current economic activity	Full-time employment	2	143	1.4
	Part-time employment	1	88	1.1
	Non-work	1	49	2.0
	Sickness/disability	0	17	0.0
	Other	0	9	0.0
General state of health	Excellent	1	50	2.0
	Very good	2	94	2.1
	Good	1	92	1.1
	Fair	0	48	0.0
	Poor	0	22	0.0
BMI classification	Underweight	0	12	0.0
	Healthy weight	3	92	3.2
	Overweight	0	94	0.0
	Obese	1	89	1.1
	Morbidly obese	0	19	0.0
Total malaise score (grouped)	Low	4	228	1.7
	High	0	78	-
Warwick–Edinburgh mental wellbeing scale	Mean	51.75	48.79	-

**Table 5:** Post hoc comparison of carers and non-carers meeting the UK chief medical officers’ physical activity guidelines.

Guideline	Carers Meeting Guideline (*n* (%))	Non-Carers Meeting Guideline (*n* (%))	Carers Not Meeting Guideline (*n* (%))	Non-Carers Not Meeting Guideline (*n* (%))
MVPA minutes	7 (2.3%)	106 (4.2%)	303 (97.7%)	2445 (95.8%)
Muscle strengthening	81 (26.1%)	632 (24.8%)	229 (73.9%)	1919 (75.2%)
Overall	4 (1.3%)	37 (1.5%)	306 (98.7%)	2514 (98.5%)

## Data Availability

The data that support the findings of this study are available from https://www.ukdataservice.ac.uk/ (accessed on 10 October 2024). Restrictions apply to the availability of these data, which were used under licence for this study.

## Abbreviations

The following abbreviations are used in this manuscript:

PA	Physical Activity
SB	Sedentary Behaviour
BCS70	1970 British Cohort Study
NHS	National Health Service
MVPA	Moderate-to-Vigorous Physical Activity
UK CMO	UK Chief Medical Officer
ANCOVA	Analysis of Covariance
BMI	Body Mass Index
WHO	World Health Organisation
UK	United Kingdom
GB	Great Britain
IPAQ	International Physical Activity Questionnaire
PASE	Physical Activity Scale for the Elderly
RAPA	Rapid Assessment of Physical Activity
